# Impedance Spectroscopy Analysis of Field-Assisted Sintered Sr- and Mg-Doped Lanthanum Gallate

**DOI:** 10.3390/ma19163481

**Published:** 2026-08-18

**Authors:** Shirley L. Reis, Cyrile F. N. Gonin, Thiago N. Machado, Marcos A. C. Berton, Reginaldo Muccillo, Eliana N. S. Muccillo

**Affiliations:** 1 Center of Science and Technology of Materials, Energy and Nuclear Research Institute, São Paulo 05508-000, SP, Brazil; shirley.shirleyreis@gmail.com (S.L.R.); muccillo@usp.br (R.M.); 2 Institute for Innovation in Electrochemistry-SENAI, Curitiba 80215-090, PR, Brazil; cyrille.gonin@sistemafiep.org.br (C.F.N.G.); thiago.machado@sistemafiep.org.br (T.N.M.); marcos.berton@sistemafiep.org.br (M.A.C.B.)

**Keywords:** lanthanum gallate, impedance spectroscopy, dopants, microstructure

## Abstract

In this study, the relationship between the microstructure and electrical conductivity of doped lanthanum gallate was investigated to identify the origin of the relatively high resistivity of the grain boundaries in this ceramic solid electrolyte. LaGaO_3_ containing acceptor dopants, Sr and Mg, was chemically synthesized and consolidated by field-assisted sintering technology. The relative density achieved 98% upon sintering at 1200 °C, and no intragrain porosity was found. The microstructure consisted of submicron-sized grains and exhibited a predominantly transgranular fracture mode. Structural characterization evidenced that all sintered samples display the characteristic orthorhombic crystal structure. Rietveld analysis revealed a secondary phase content of only ~0.71% in samples sintered at 1200 °C. In addition, Raman spectra revealed only the allowed characteristic vibrational modes expected for doped lanthanum gallate. The electrical conductivity was determined by impedance spectroscopy analysis. The bulk conductivity of sintered samples was found to be independent of the sintering temperature. Analysis of the grain boundary resistivity revealed a dependence on the mean grain size, which constricts the pathway of the charge carriers, leading to the formation of space charge layers. The total activation energy determined for conduction is 0.94 eV.

## 1. Introduction

Oxygen ion conductors have been investigated for applications in electrochemical devices such as sensors, solid oxide fuel cells (SOFCs), and oxygen pumps [1,2]. Conventional SOFCs utilize zirconia containing 8 to 9 mol% yttria as the solid electrolyte and operate in the 800 to 1000 °C range [3,4]. At such high temperatures, the electrochemical reactions are accelerated, leading to increased power output and high current density, as well as allowing for fuel flexibility and reforming. However, the high temperatures utilized are also responsible for high degradation rates and increased costs due to the utilization of expensive materials [3,4,5].

Over the last three decades, intensive research work has been carried out to lower the operating temperature of SOFCs, aiming to reduce the cell degradation rate and, therefore, increase the benefit-to-cost ratio, with a view to its commercialization. One approach to overcome the deleterious effects imposed by the operating temperature is the use of a solid electrolyte with higher ionic conductivity than stabilized zirconia.

Owing to its high ionic conductivity, approximately 2.5 times that of yttria-stabilized zirconia at the same temperature, doped lanthanum gallate containing partial substitutions in the A- and B-sites of the perovskite structure for Sr and Mg, respectively, has been thoroughly investigated for SOFC operation in the so-called intermediate temperature range (600 to 800 °C) [6,7]. The introduction of acceptor cations, such as Sr^++^ and Mg^++^, into the LaGaO_3_ structure promotes the creation of oxygen vacancies for charge compensation according to Equation (1):

(1) $$SrO+MgO\overset{LaGaO_{3}}{\to }{Sr}_{La}^{\prime }+{Mg}_{Ga}^{\prime }+2O_{O}^{x}+V_{Ö}$$

Several compositions have been studied, and it is well recognized that higher ionic conductivities are obtained when 0.1 ≤ x, y ≤ 0.2 in La_1-x_Sr_x_Ga_1-y_Mg_y_O_3-δ_. Furthermore, the crystalline structure of doped lanthanum gallate changes with dopant content from orthorhombic (x + y ≤ 0.25) to a mixture of orthorhombic and rhombohedral (0.25 ≤ x + y ≤ 0.30), becoming cubic for x + y ≥ 0.35 [8]. In addition to the high ionic conductivity, doped lanthanum gallate exhibits a wide electrolytic domain (1 to 10^−22^ atm at 800 °C), suitable mechanical properties, and good stability, even at 700 °C in a hydrogen atmosphere [6,7,9,10]. Recently, it has been shown that the combination of its properties turns this ceramic material into a promising solid electrolyte in SOFCs for energy production and green hydrogen generation in solid oxide electrolysis cells (SOECs) [11,12,13].

Doped lanthanum gallates are in general prepared by the solid-state synthesis method, a simple and cost-effective method that integrates high production rate and low cost. The complex composition of doped lanthanum gallate accounts for some concerns, such as the frequently observed secondary phases and its relatively low sinterability. The secondary phases found in specimens prepared by the solid-state synthesis method are: La_4_Ga_2_O_9_, LaSrGaO_4_, LaSrGa_3_O_7_ and MgO [14]. These secondary phases exhibit low ionic conductivity compared to that of doped lanthanum gallate and are believed to be formed from Ga loss due to the high temperatures used during sample preparation and to the solubility and contents of co-dopants [15,16]. Among the compositions with high ionic conductivity, special attention has been given to La_0.9_Sr_0.1_Ga_0.8_Mg_0.2_O_3-δ_, hereafter referred to as LSGM, because of its comparatively low content (~5%) of secondary phases. The ionic conductivity of this composition was found to amount 0.127 S.cm^−1^ at 800 °C [17].

Apart from the traditional solid-state method for synthesizing the complex composition of LSGM, solution methods have been used such as coprecipitation, sol-gel, spray pyrolysis and combustion [18,19,20,21]. As a rule, the LSGM powders obtained by these chemical methods are of high purity, with homogeneous distribution of the cations, and high specific surface area. Comparatively low fractions (≤5%) of secondary phases were obtained for chemically synthesized powders depending on specific protocols used during specimens manufacturing. Nevertheless, temperatures above 1300 °C are generally required to achieve high densification. The low sinterability of doped lanthanum gallates is, to some extent, attributed to the solubility of the acceptor cations (Sr^2+^ and Mg^2+^) in LaGaO_3_, which is maximum at 1500 °C [22]. Nevertheless, the high temperatures and long dwell times required in conventional sintering contribute to elemental diffusion, increasing the fraction of secondary phases.

The technological applications of doped lanthanum gallates require dense ceramic specimens. Then, the sintering of this solid electrolyte has received great attention. In this context, utilization of sintering aids [23,24,25], the use of controlled atmosphere [26] and diverse sintering methodologies like fast, microwave and two-stage sintering [27,28,29] have been investigated. Regarding sintering techniques applying simultaneously temperature and pressure, hot isostatic pressing [30] and field-assisted sintering technology, also known as spark plasma sintering, SPS [31,32,33,34] have proven to be highly effective to generate high-density specimens at relatively low temperatures.

Concerning the LSGM ceramics obtained by SPS, the starting powders were chemically prepared by glycine-nitrate combustion [31], Pechini [32] and precipitation followed by hydrothermal methods [33]. For powders prepared by the combustion method and sintered at 1300 °C, the density achieved 94.7% of the theoretical value. The mean grain size was less than 1 µm [31]. Application of high pressures (~700 MPa) during SPS in LSGM powders prepared by the Pechini method allowed for obtaining near full density at relatively low temperatures, with grain sizes in the nanometric range [32]. LSGM prepared by precipitation and hydrothermal methods and spark plasma sintered at 1200 °C exhibited relative densities higher than 90% [33]. In these previous reports, the fraction of secondary phases was about 3%. These studies focused on the effect of the grain size on the magnitude of the ionic conductivity. Recently, the effect of the grain size on the electronic *p*-type conductivity of LSGM was studied on specimens synthesized by a modified Pechini method [34].

In ceramic materials with grain sizes with low dimensions (below 1 µm), the influence of the grain boundaries on the ionic conductivity is of prime relevance, because the interfaces are responsible for a blocking effect to the transport of the charge carriers (oxygen ions). Then, in this work, chemically synthesized LSGM powder was sintered by the field-assisted sintering technology and characterized by structural, microstructural and electrochemical impedance spectroscopy, aiming to clarify the role of the bulk and the grain boundaries on the total ionic conductivity of this solid electrolyte. Other purposes were to minimize the fraction of impurity phases and to identify the origin of the blocking effect at the grain boundaries and interfaces in LSGM.

## 2. Materials and Methods

### 2.1. Materials Preparation

Lanthanum nitrate (99.99%, Alfa Aesar, Ward Hill, MA, USA), strontium carbonate (99%, Sigma Aldrich, St. Louis, MO, USA), gallium nitrate (99.99%, Alfa Aesar) and magnesium nitrate (99%, Sigma Aldrich) were the starting materials. The nominal composition La_0.9_Sr_0.1_Ga_0.8_Mg_0.2_O_3-x_ was prepared by the complexation method as fully described in [35]. In brief, water solutions of the precursors were mixed in the stoichiometric proportion under vigorous stirring and heating with a water solution of citric acid. After drying, the collected porous foam was heat treated at 250 °C. The nanosized powder was subsequently calcined at 800 °C for 1 h and field assisted sintered (Thermal Technology, LCC SPS model 10-4, Minden, NV, USA).

In a typical experiment, the nanosized powder was loaded into a graphite die with graphite foils (bottom and cover) and with graphite punches. The whole set of die and punches was heated at a rate of approximately 100 °C.min^−1^ under vacuum with 60 MPa of pressure to the dwell temperature: 1100, 1150 and 1200 °C. After 5 min of dwell time, the electric current was switched off. Then, cooling down proceeded to room temperature, when the applied pressure was released. The surfaces of sintered samples were sanded to the final dimensions: ~9.7 mm of diameter and ~1 mm thickness.

### 2.2. Characterization

Characterization of the synthesized nanostructured powder may be found in a previous publication [35].

The density of sintered samples was measured using the Archimedes method. The theoretical density of 6.67 g.cm^−3^ from ICSD 51-288 was used to calculate the relative density.

Microstructural characterization was performed by field-emission gun scanning electron microscopy and SEM (FEI, inspect F50, Brno, Czech Republic) in both fractured surfaces and polished and thermally etched surfaces followed by carbon coating. Energy dispersive spectroscopy, EDS, was utilized for elemental mapping of sintered samples.

The crystalline structure was studied by X-ray diffraction, XRD (Bruker D2 Phaser, Billerica, MA, USA), in the 10° ≤ 2θ ≤ 90° range, with Cu K_α_ radiation (λ = 1.5405 Å), with 40 kV and 30 mA, 0.02° step size and 2 s of counting time. The phase purity of the sample sintered at 1200 °C was evaluated by Rietveld analysis (Topas, version 5.0). Additional structural characterization was performed by Raman spectroscopy (Qontor, in Via^TM^ Raman microscope, Renishaw, Gloucestershire, UK) with a 532 nm laser, 50 mW of power and 10 s of acquisition time.

The sintered samples were spring loaded between platinum disks inside a custom-made inconel 600 sample-chamber connected to the impedance analyzer. Electrical measurements were carried out by impedance spectroscopy (HP 4192A, LF impedance analyzer, connected to a 362-model controller, Yokogawa, Tokyo, Japan), with 100 mV of input signal and 20 points per decade in the 10 Hz to 10 MHz and 130 to 300 °C frequency and temperature ranges, respectively. The measuring temperature was monitored with a chromel–alumel (K-type) thermocouple, with its tip positioned close to the samples. The measurements were performed after 55 min of equilibration time at each temperature. Gold was applied by sputtering onto large surfaces of samples for electrical measurements.

## 3. Results and Discussion

### 3.1. Structure and Microstructure

All the LSGM samples submitted to SPS achieved high relative density, as listed in Table 1. The high densification level obtained after short dwell time indicates that the synthesized powder consisted predominantly of soft agglomerates, which facilitate mass transport during consolidation.

The relative density increased from 93% to 98% with increasing sintering temperature, accompanied by moderate grain growth. Despite the increase in grain size, all specimens retained a fine-grained microstructure with average grain sizes smaller than 1 μm.

**Figure 1:**
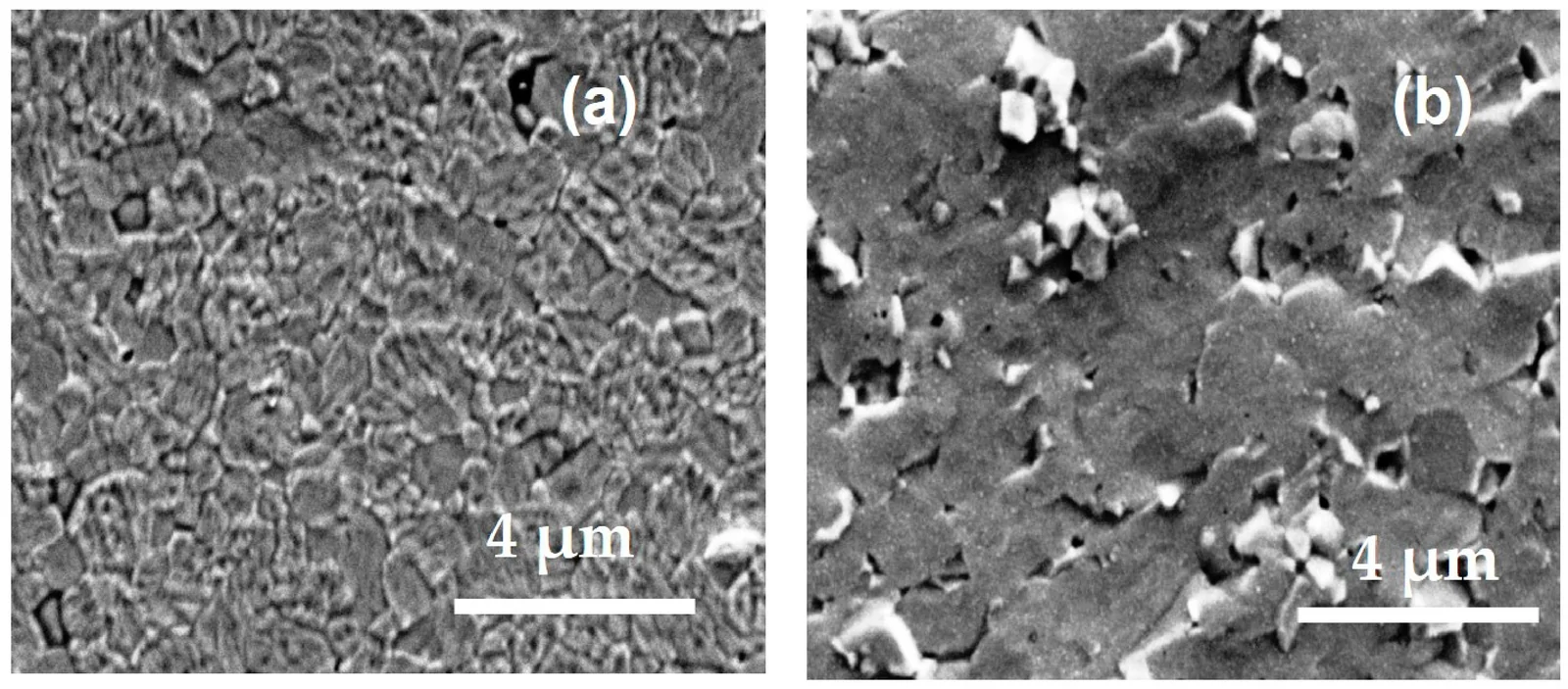
SEM micrographs of LSGM sintered at 1200 °C. (**a**) Polished and (**b**) fractured surfaces.

Representative SEM micrographs of the sample sintered at 1200 °C are shown in Figure 1. The polished and thermally etched surface (Figure 1a) reveals a dense and homogeneous microstructure consisting of grains in the submicron range. Few residual pores are found at triple grain junctions, and no intragrain porosity is observed. The fracture surface (Figure 1b) exhibits a predominantly transgranular fracture mode. It is worth noting that this result is typical of materials free from amorphous phases, which weakens the grain boundaries and, consequently, may reduce the mechanical strength of the ceramic material. The microstructure of samples sintered at 1150 and 1100 °C were qualitatively similar, except that, with decreasing sintering temperature, increased porosity and decreased grain size were found. The mean grain size, *d_g_*, listed in Table 1, evidences the submicron range in sintered samples.

**Figure 2:**
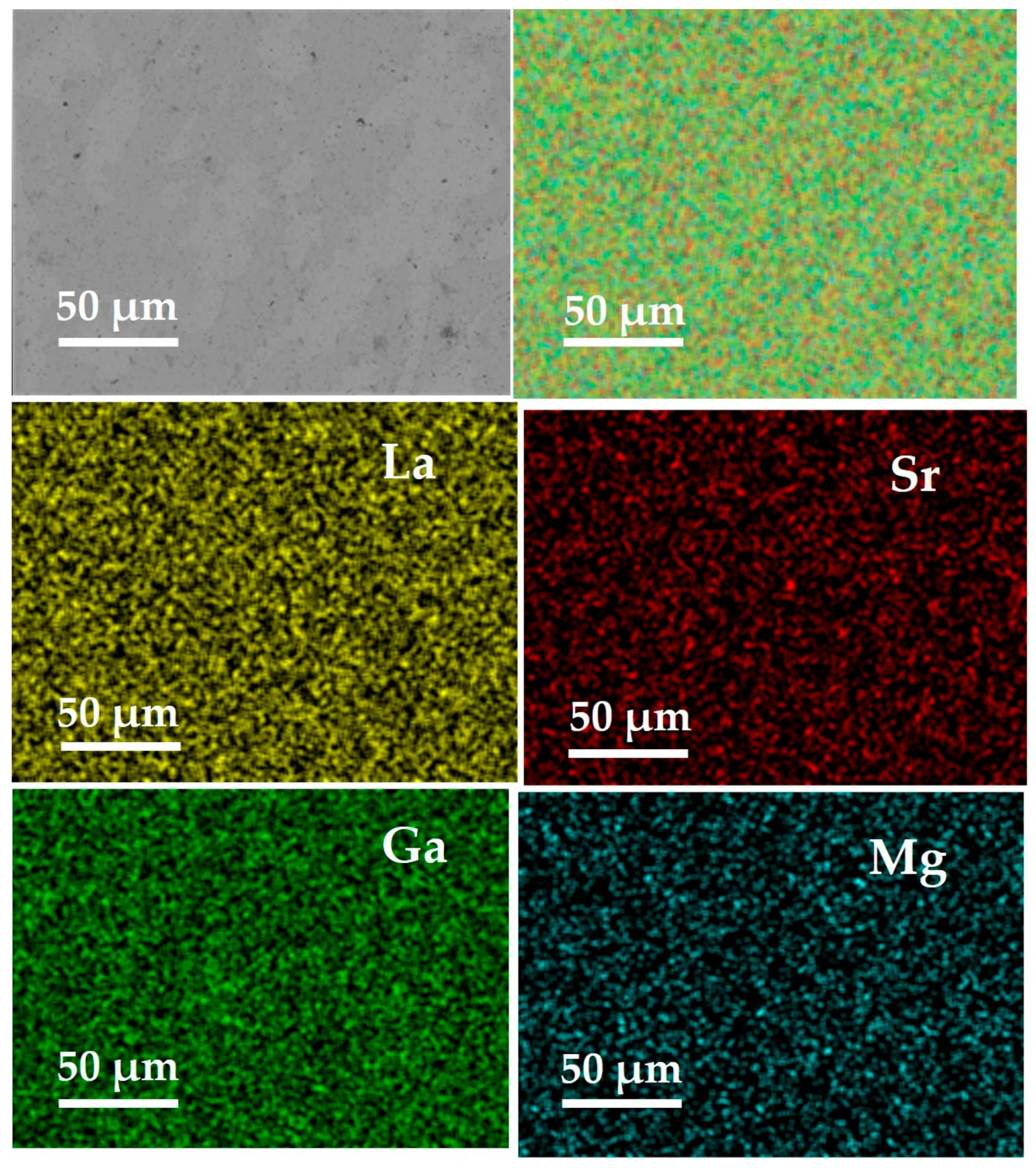
Backscattered image of the sample sintered at 1100 °C (**top left**), general elemental mapping of the sample cations (**top right**) and individual elemental maps for La (yellow), Sr (red), Ga (green) and Mg (blue). Bar length = 50 µm.

Figure 2 shows, as example, the images obtained with EDS for LSGM sintered at 1100 °C. The backscattered image of a selected microregion is shown in the top left-side corner, while the combined elemental map is displayed in the top right-side corner. Individual elemental distributions corresponding to La (yellow), Sr (red), Ga (green) and Mg (blue) are also shown. The combined elemental map indicates a homogeneous distribution of all constituent cations throughout the analyzed microregion. Furthermore, the individual elemental maps reveal no evidence of elemental clustering within the spatial resolution of the technique. Similar results were obtained for the other sintered specimens. These observations demonstrate that the homogeneous cation distribution achieved during chemical synthesis was preserved during consolidation.

**Figure 3:**
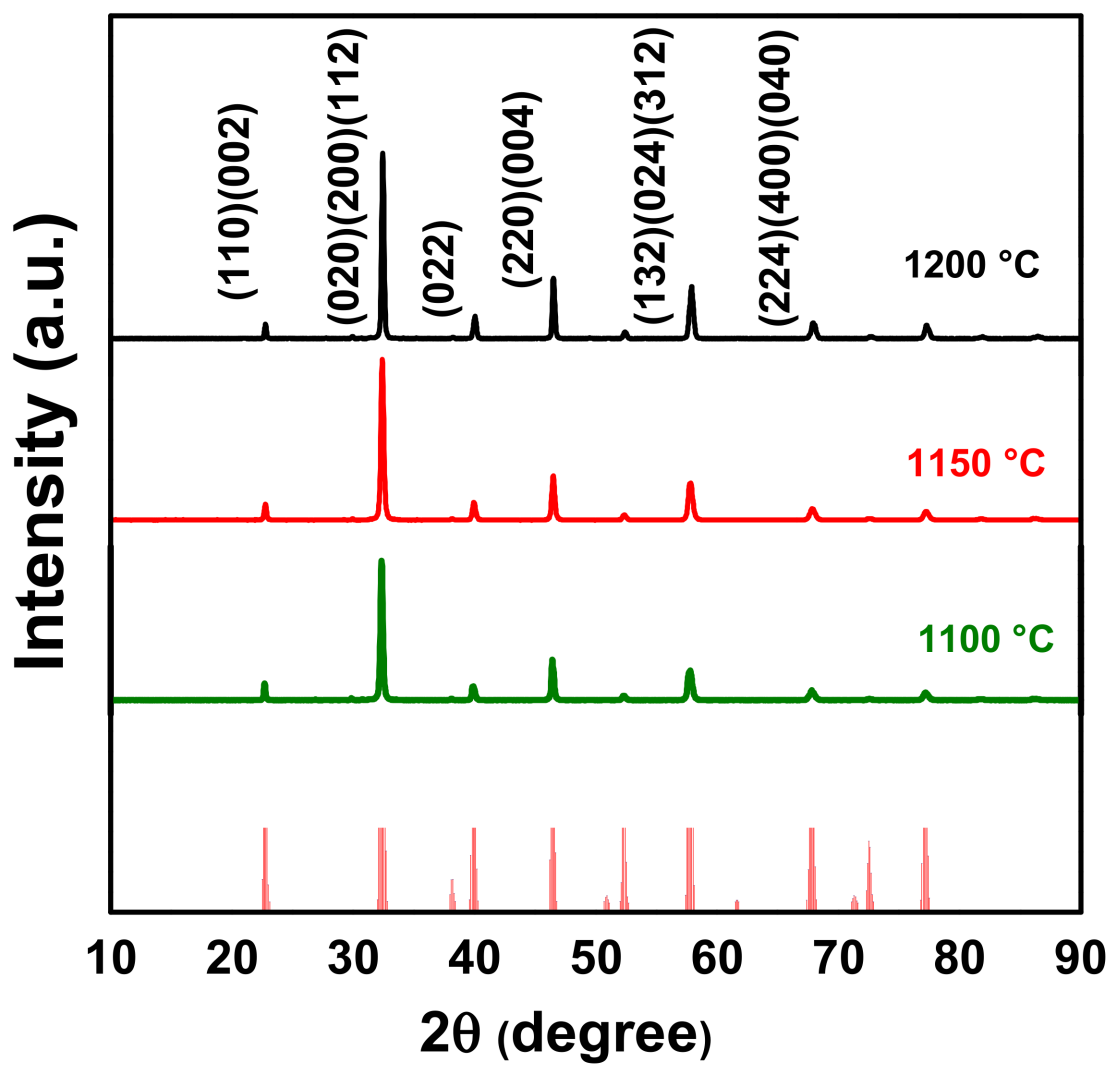
XRD patterns of sintered LSGM samples. Red tick marks at bottom are the main reflections of the orthorhombic structure.

Figure 3 depicts the room temperature XRD patterns of sintered LSGM samples sintered at different temperatures. The main diffraction peaks of the orthorhombic structure are shown in the bottom by the red thick marks, and only a few peaks are indexed in this figure for clarity.

**Figure 4:**
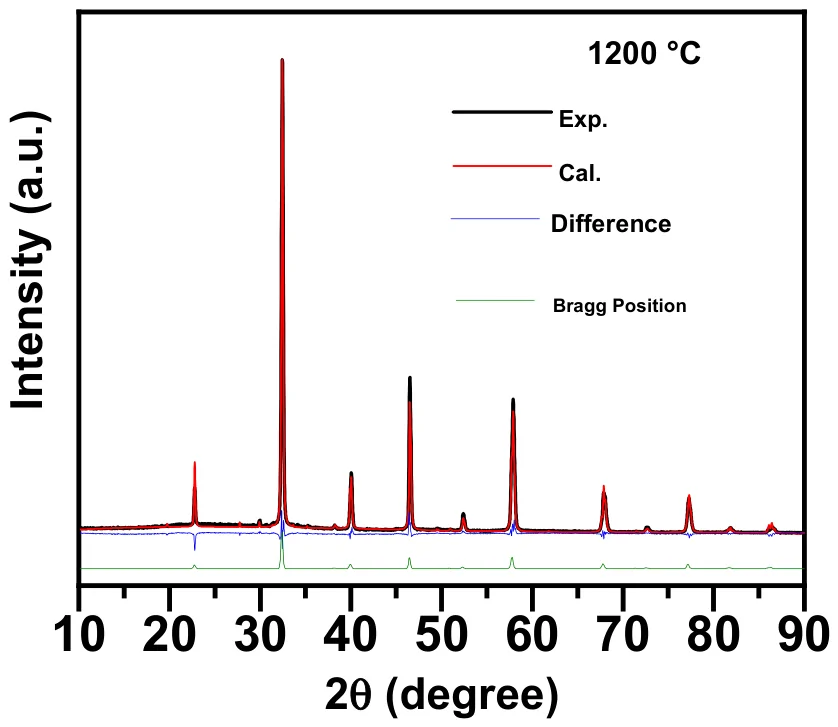
XRD pattern and Rietveld analysis of LSGM sintered at 1200 °C.

The LSGM sintered with different dwell temperatures display orthorhombic structure. No diffraction peaks associated with secondary phases were detected within the resolution limits of conventional XRD measurements. To further investigate phase purity and crystal structure, Rietveld refinement was performed for the sample sintered at 1200 °C. The experimental and calculated diffraction patterns are shown in Figure 4.

The best fit was obtained using an orthorhombic structural model belonging to the Imma space group, with refined lattice parameters of a = 5.5356(9) Å, b = 7.8001(6) Å, and c = 5.5090(1) Å. The refinement quality indicators were Rexp = 2.97, Rwp = 11.13, Rp = 8.35, and GOF = 3.75, confirming the adequacy of the structural model. Quantitative phase analysis revealed the presence of approximately 0.71 wt.% LaSrGa_3_O_7_ as a secondary phase. This amount is considerably lower than values commonly reported for conventionally processed LSGM ceramics, demonstrating the effectiveness of the chemical synthesis route combined with SPS consolidation in suppressing secondary-phase formation.

**Figure 5:**
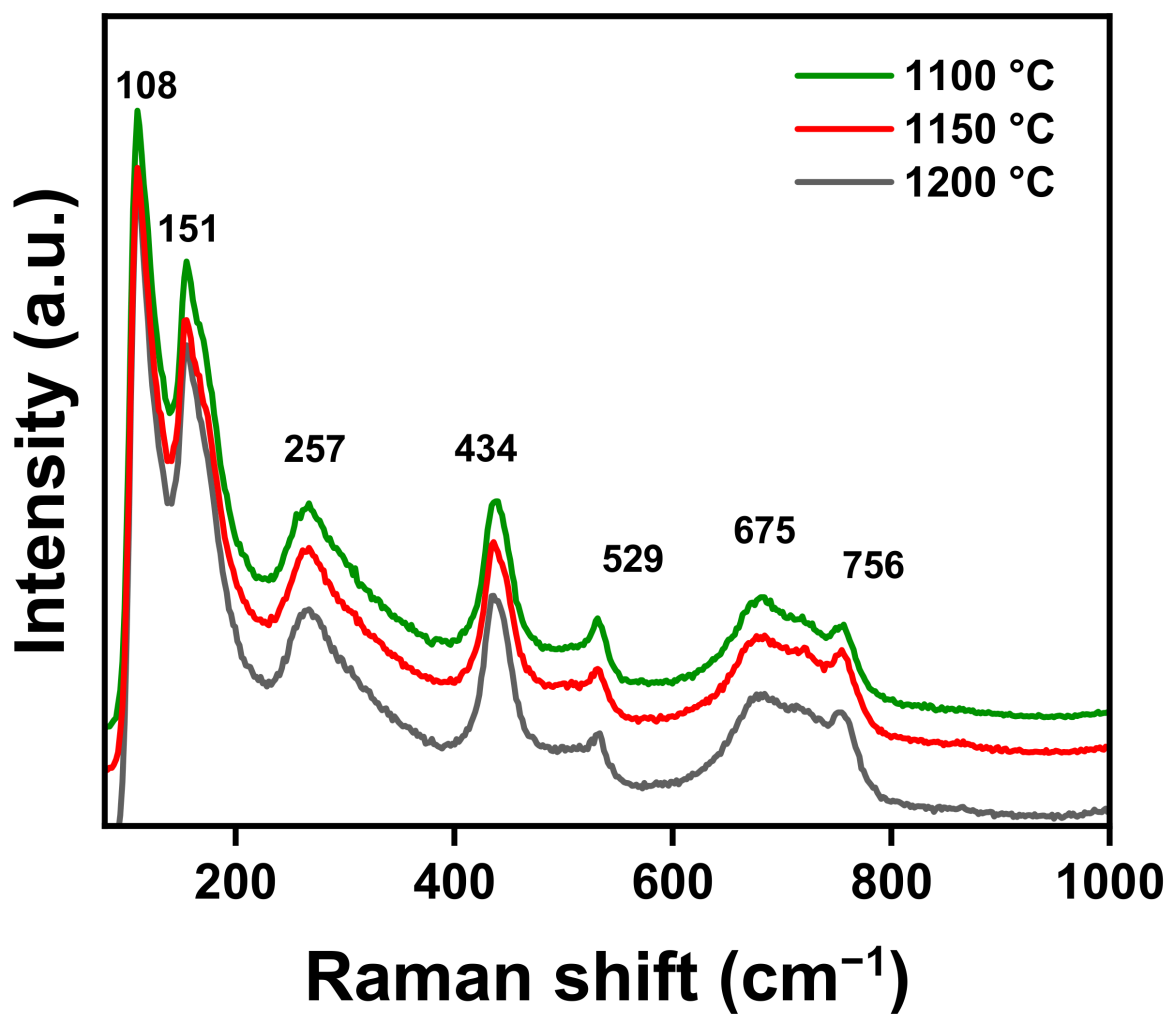
Raman spectra of sintered LSGM samples.

Room temperature Raman spectra of sintered LSGM samples are shown in Figure 5. Previous studies have shown that orthorhombic LSGM exhibits several Raman bands in the 100 to 800 cm^−1^ spectral range [36,37]. The Raman bands below 200 cm^−1^ are assigned to vibrations in the A-site cations in the perovskite structure. In the 200 to 300 cm^−1^ range, a single band at 257 cm^−1^ is observed. This band is attributed to GaO_6_ octahedra tilt. The active mode at 434 cm^−1^ is due to out-of-phase bending vibration. For the three active modes in 480 to 800 cm^−1^, in this case, the bands with maximum amplitudes at 529, 675 and 756 cm^−1^ were shown to be related to the amounts of Sr and Mg added to LaGaO_3_. Increasing the fractions of Sr and Mg results in widening and decreasing intensity of these Raman bands. Then, these Raman bands come up from the decrease in the local symmetry owing to the oxygen vacancies generated by doping according to Equation (1) [36].

The results demonstrate effective densification at a reduced sintering temperature (1200 °C), yielding a sub-micrometer mean grain size, whereas conventional sintering (1400–1500 °C) produces coarser grains (2–5 µm) [28,29]. Additionally, the secondary phase fraction (0.71%) remains significantly lower than typical literature values [10,17,31,32]. Overall, the structural and microstructural characterization results demonstrate that the combination of chemical synthesis and SPS consolidation produced highly dense and chemically homogeneous LSGM ceramics with submicron grain sizes and a very low concentration of secondary phase. 

### 3.2. Impedance Spectroscopy

**Figure 6:**
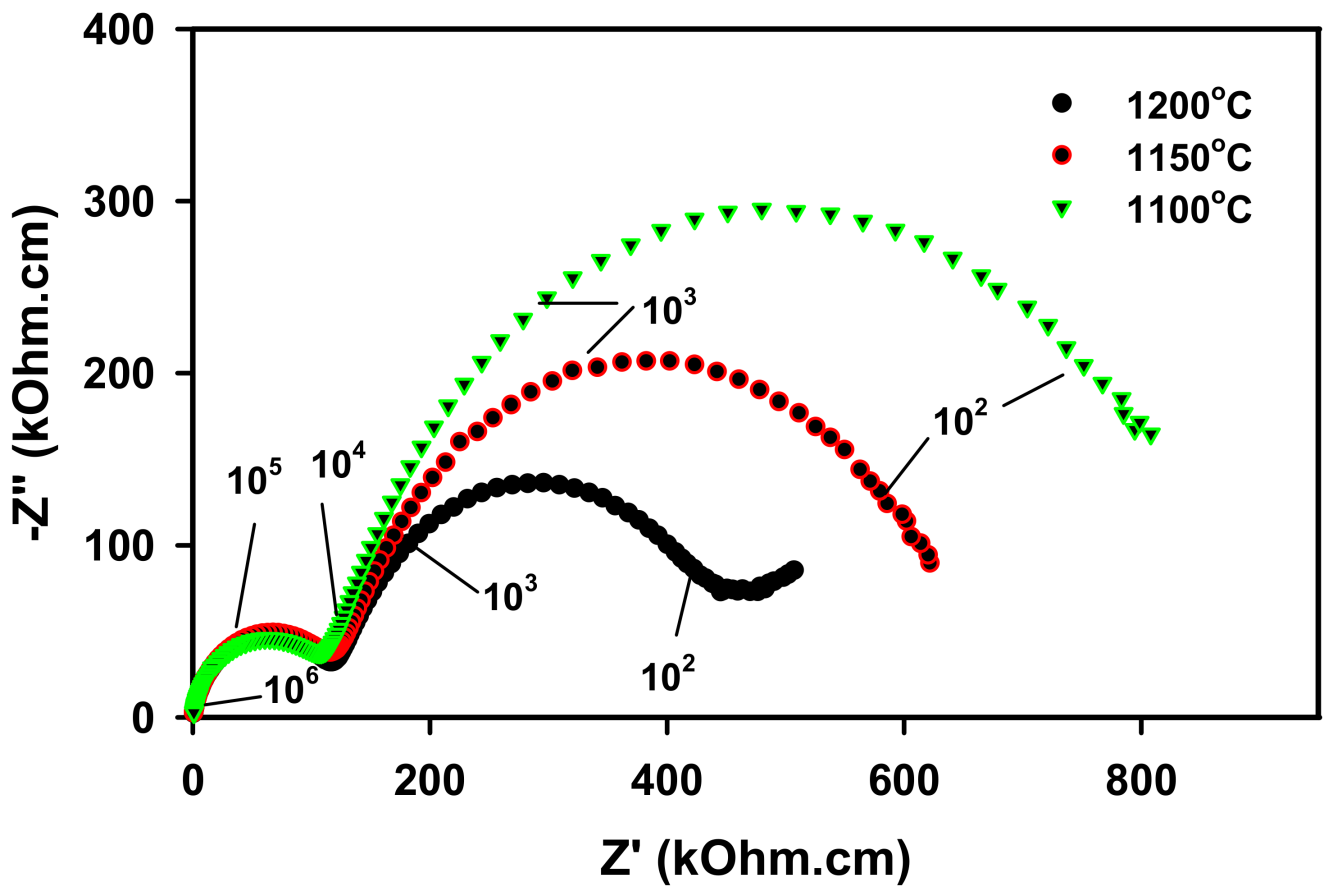
−Z″ (ω) × Z′ (ω) Nyquist plots of sintered LSGM. Temperature of measurement = 225 °C.

Figure 6 depicts −Z″(ω) × Z′(ω) plots of sintered LSGM samples obtained at 225 °C. The complex (Z″) and the real (Z′) components of the total impedance were normalized for sample dimensions for comparison. The numbers are the relaxation frequencies, in Hz. The high-frequency arc is attributed to the bulk response of the materials, whereas the intermediate-frequency arc corresponds to the grain boundary contribution. The spike observed at frequencies lower than 10^2^ Hz in the sample sintered at 1200 °C is due to the reactions occurring at the sample–electrode interface and becomes noticeable with increasing temperature. Deconvolution of the diagrams was carried out, assuming an equivalent electrical circuit consisting of two sets (one for the bulk and the other for the grain boundaries and interfaces) connected in series to a parallel resistor-constant phase element network. The evolution of the electrical conductivity, σ, with the measuring temperature was assessed by the Arrhenius expression:

(2) $$\sigma =\frac{\sigma_{O}}{T}exp(-\frac{E}{kT})$$

where $\sigma_{0}$, *E*, *k* and *T* are, respectively, the pre-exponential factor, the activation energy for conduction, the Boltzmann constant and the absolute temperature.

**Figure 7:**
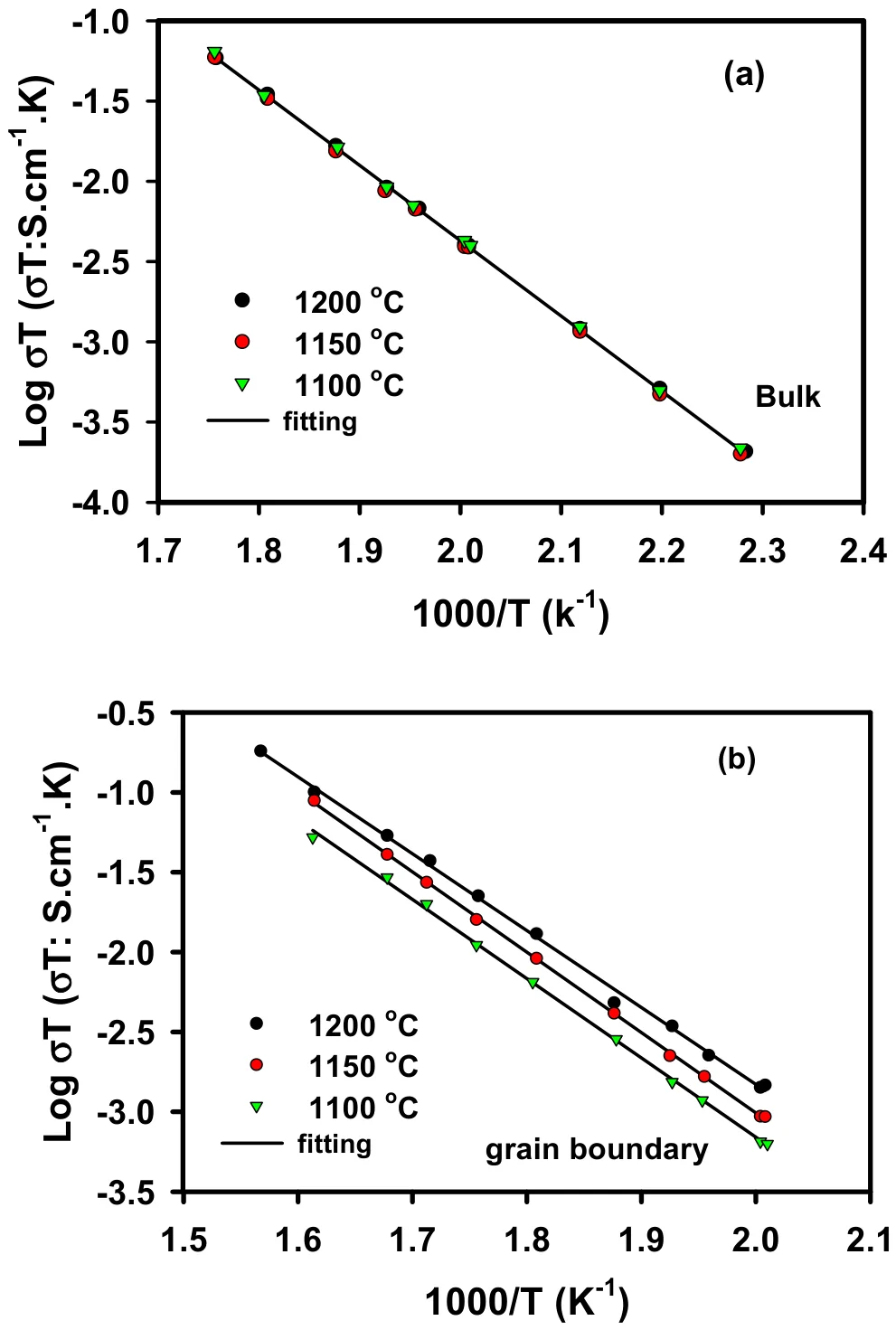
Arrhenius plots of the electrical conductivity of sintered LSGM samples. (**a**) Bulk and (**b**) grain boundaries.

The high-frequency arc did not experience any significant change with the sintering temperature. This is seen in Figure 6 and is highlighted in the Arrhenius plots of the bulk conductivity in Figure 7a. A single straight line fitted all the experimental data. This means that statistically there are no changes in the stoichiometry of the bulk of LSGM samples with the sintering temperature. The calculated activation energy for bulk conduction is 0.93 ± 0.05 eV. This result suggests that the concentration and mobility of oxygen ions within the grains were not affected by the sintering process.

Despite the good distribution of the cations in chemically synthesized powders, minor variations in the composition are expected to occur during sintering. This effect is due to an attractive Coulomb force between the dopant cation and the oxygen vacancy, forming associates. Atomistic simulations have shown that the association of magnesium ions with oxygen vacancies at nearest-neighbor sites (${Mg}_{Ga}^{\prime }V_{Ö}{Mg}_{Ga}^{\prime }$)*^x^* is energetically favorable in LSGM [38]. Then, in addition to the long-range migration of free oxygen vacancies introduced by doping, the motion of a bound vacancy among all its equivalent positions in an associate may contribute to the activation energy value. The magnitude of the localized transport of oxygen ions in associates is very small and contributes only marginally to the measured conductivity. The activation energy of bound vacancies in LSGM was reported to be only 0.06 eV [39].

In contrast to the bulk electrical response, the grain boundary contribution exhibited a clear dependence on sintering temperature. As shown in Figure 6, the diameter of the grain boundary arc progressively decreased with increasing sintering temperature. The Arrhenius plots of grain boundary conductivity (Figure 7b) confirm this behavior, revealing a systematic increase in grain boundary conductivity as the mean grain size increased. The corresponding activation energy values (E_gb_) are slightly higher than those of the bulk and are ~1 eV.

**Figure 8:**
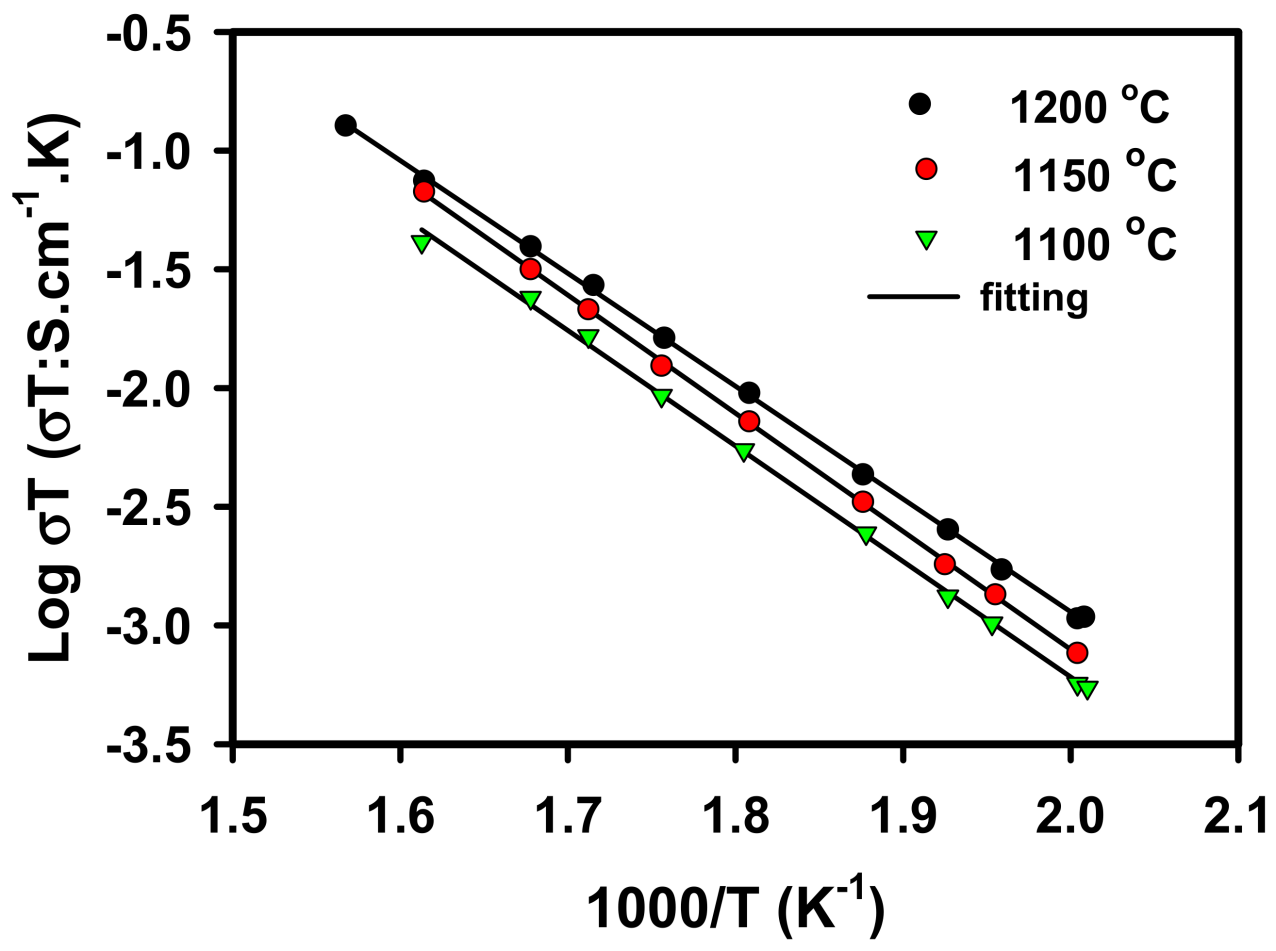
Arrhenius plots of the total (grain plus grain boundaries) electrical conductivity of sintered LSGM samples.

The total electrical conductivity, including both bulk and grain boundary contributions, is shown in Figure 8. Linear Arrhenius behavior was observed throughout the investigated temperature range, yielding an activation energy of 0.94 ± 0.05 eV.

The relatively high grain boundary resistivity observed in oxide-ion conductors may originate from either intrinsic or extrinsic effects. Intrinsic effects have their origin due to imperfect grain boundaries, defects in the crystal lattice such as stacking faults and dislocations, or accumulation of the dopants at or near the grain boundaries. Stacking faults may induce intrinsic blocking effects in ceramics by creating localized structural and energetic barriers that impede or slow down the movement of charge carriers. On the other hand, the extrinsic effects are mainly caused by impurities such as those existing in the starting materials or intentionally added like sintering aids. These features, either intrinsic or extrinsic, account for a blocking effect at the grain boundaries for diffusion of the charge carriers, as well as the formation of space charge layers. To identify the origin (intrinsic or extrinsic) of the grain boundary resistivity in sintered LSGM, the model proposed by Miyayama and Yanagida [40] was employed.

According to this approach, the grain boundary resistance per unit surface area of the grain boundaries, $R_{sgb}$, is related to the mean grain size, *d_g_*, and the measured grain boundary resistance, $R_{gb}$, by:

(3) $$R_{sgb}=R_{gb}{\times d}_{g}$$

Afterward, normalization of the resistance values to unit cross-sectional area and unit length is carried out for all sintered samples obtaining the corresponding specific grain boundary resistivities ($\rho_{sgb}$).

In the absence of extrinsic effects, the specific resistivity of grain boundaries should not change with the mean grain size, provided that it is only the grain boundary area that changes in the sample as a function of the mean grain size. In contrast, if extrinsic effects dominate the grain boundary electrical response, the specific resistivity should increase with increasing mean grain size [40].

**Figure 9:**
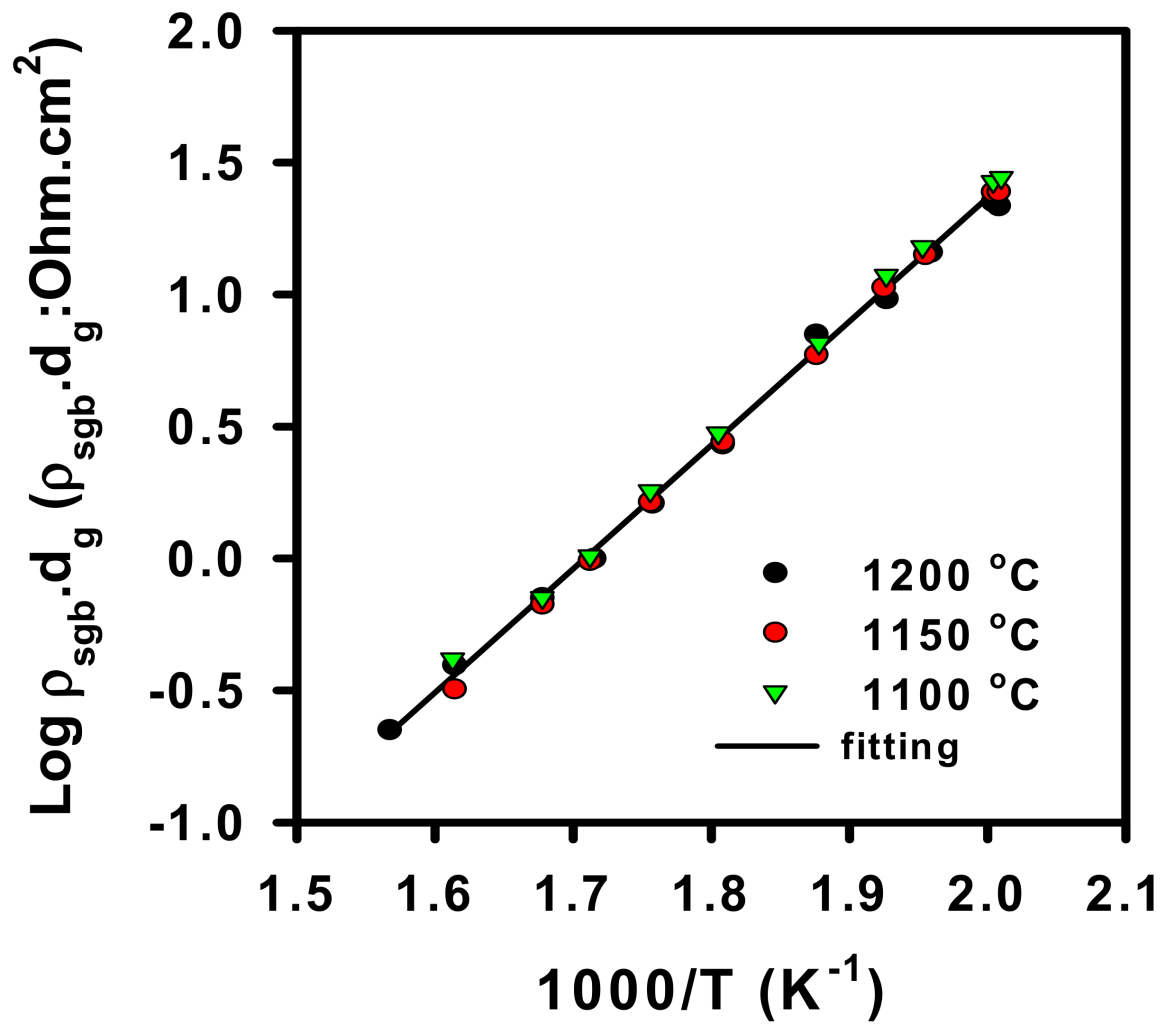
Temperature dependence of the specific electrical resistivity of the grain boundaries of sintered LSGM samples.

Figure 9 shows Arrhenius plots of the calculated specific grain boundary resistivity for sintered samples. Remarkably, all data can be described by a single straight line, evidencing that the grain boundary resistivity of LSGM samples depends only on the mean grain size, thereby characterizing an intrinsic blocking effect. A possible reason for the intrinsic blocking effect in LSGM is the existence of stacking faults, observed by transmission electron microscopy in this ceramic material [37]. A rigorous analysis by transmission electron microscopy could clarify its influence in this case. Although Rietveld refinement detected a small amount (0.71 wt.%) of LaSrGa_3_O_7_, its concentration is considerably lower than those typically reported for conventionally sintered LSGM ceramics and is unlikely to be the dominant source of the observed grain-boundary blocking effect.

## 4. Conclusions

The overall results evidence that homogeneous LSGM powder was successfully synthesized by chemical method. After sintering by SPS technology in the 1100 to 1200 °C range, the consolidated samples achieved high densities (93 to 98% of the theoretical value), with mean grain sizes in the submicron range (350 to 640 nm). Rietveld refinement revealed only a minor amount (0.71 wt.%) of LaSrGa_3_O_7_ secondary phase, demonstrating that the selected processing route effectively minimized secondary phase formation. Impedance spectroscopy technique was utilized to determine the origin of the blocking effect at the grain boundaries in sintered samples. The specific grain boundary resistivity remained independent of the mean grain size, indicating that the blocking effect is predominantly intrinsic in nature. The activation energy for total electrical conductivity was found to be 0.94 ± 0.05 eV. Additional research on long-term stability at usual operating temperatures and mechanical properties would be able to demonstrate the feasibility of this solid electrolyte for electrochemical applications. This study highlights the capability of impedance spectroscopy to establish direct correlations between microstructural characteristics and ionic transport mechanisms in oxide-ion conducting ceramics.

## Figures and Tables

**Table 1:** Values of relative density (ρ_r_) and mean grain size (d_g_) of sintered LSGM samples.

Sintering Temperature (°C)	ρ_r_ (±1%)	d_g_ (µm)
1100	93	0.35 ± 0.06
1150	95	0.46 ± 0.26
1200	98	0.64 ± 0.04

## Data Availability

The original contributions presented in this study are included in the article. Further inquiries can be directed to the corresponding author.
